# A novel small molecule, CU05-1189, targeting the pleckstrin homology domain of PDK1 suppresses VEGF-mediated angiogenesis and tumor growth by blocking the Akt signaling pathway

**DOI:** 10.3389/fphar.2023.1275749

**Published:** 2023-11-16

**Authors:** Jeongeun Park, Haiying Zhang, Hyun Jung Kwak, Changdev Gorakshnath Gadhe, Yeomyeong Kim, Hyejeong Kim, Minyoung Noh, Dongyun Shin, Sang-Jun Ha, Young-Guen Kwon

**Affiliations:** ^1^ Department of Biochemistry, College of Life Science and Biotechnology, Yonsei University, Seoul, Republic of Korea; ^2^ Department of Bio Research, Curacle Co., Ltd., Seoul, Republic of Korea; ^3^ Department of Strategic Planning, Curacle Co., Ltd., Seoul, Republic of Korea; ^4^ Department of New Drug Research Development, Curacle Co., Ltd., Seoul, Republic of Korea; ^5^ College of Pharmacy, Gachon University, Incheon, Republic of Korea

**Keywords:** CU05-1189, angiogenesis, PDK1, Akt, cancer therapy

## Abstract

Inhibition of angiogenesis is considered a promising therapeutic approach for cancer treatment. Our previous genetic research showed that the use of a cell-penetrating peptide to inhibit the pleckstrin homology (PH) domain of 3-phosphoinositide-dependent kinase 1 (PDK1) was a viable approach to suppress pathological angiogenesis. Herein, we synthesized and characterized a novel small molecule, CU05-1189, based on our prior study and present evidence for the first time that this compound possesses antiangiogenic properties both *in vitro* and *in vivo*. The computational analysis showed that CU05-1189 can interact with the PH domain of PDK1, and it significantly inhibited vascular endothelial growth factor (VEGF)-induced proliferation, migration, invasion, and tube formation in human umbilical vein endothelial cells without apparent toxicity. Western blot analysis revealed that the Akt signaling pathway was specifically inhibited by CU05-1189 upon VEGF stimulation, without affecting other VEGF receptor 2 downstream molecules or cytosolic substrates of PDK1, by preventing translocation of PDK1 to the plasma membrane. We also found that CU05-1189 suppressed VEGF-mediated vascular network formation in a Matrigel plug assay. More importantly, CU05-1189 had a good pharmacokinetic profile with a bioavailability of 68%. These results led to the oral administration of CU05-1189, which resulted in reduced tumor microvessel density and growth in a xenograft mouse model. Taken together, our data suggest that CU05-1189 may have great potential and be a promising lead as a novel antiangiogenic agent for cancer treatment.

## 1 Introduction

Angiogenesis is a tightly regulated process of new blood vessel formation involving proliferation, migration, invasion, and new tube formation by endothelial cells (Otrock et al., 2007). This process is critical in maintaining physiological homeostasis and regeneration (Apte et al., 2019; Jiang et al., 2020). However, under pathological conditions such as cancer, uncontrolled and excessive neovascularization can occur because malignant cells require maximum nutrient and oxygen supplies to support their continued growth (Jiang et al., 2020; Lugano et al., 2020). Therefore, because angiogenesis is differentially activated between normal and tumor cells, targeting angiogenesis has become an attractive therapeutic approach for cancer treatment (Al-Abd et al., 2017). Many different types of antiangiogenic agents have been developed, and more than 10 drugs have been approved by the Food and Drug Administration (Lugano et al., 2020). Nonetheless, most of the available drugs only provide limited benefits for certain tumor types and have several adverse effects (Rajabi and Mousa, 2017). Thus, it is essential to develop more effective and less toxic agents.

Many growth factors and cytokines are involved in the regulation of angiogenesis, but vascular endothelial growth factor (VEGF) is considered the most important regulator (Ferrara et al., 2003). VEGF can activate various signaling mediators in vascular endothelial cells, including ERK1/2 MAP kinase, FAK, p38 MAP kinase, and Akt, which coordinate to regulate the angiogenic process (Qi et al., 2022). Among these, the Akt signaling pathway predominantly orchestrates growth factor-induced angiogenic responses, with 3-phosphoinositide-dependent kinase 1 (PDK1) acting as a key mediator in this process (Shiojima and Walsh, 2002; Leroux et al., 2018). Upon growth factor stimulation, both PDK1 and Akt bind to the second messenger PI(3,4,5)P3 with strong affinity, leading to their recruitment to the plasma membrane (Leroux et al., 2018). This co-localization at the plasma membrane enables Akt phosphorylation and activation by PDK1, which in turn regulates various cellular functions including proliferation, migration, invasion, and growth (Shiojima and Walsh, 2002; Karar and Maity, 2011; Tawaramoto et al., 2012).

PDK1 is a serine/threonine kinase that belongs to the AGC kinase family and possesses two well-known functional domains, an N-terminal kinase catalytic domain and a C-terminal pleckstrin homology (PH) domain (Bayascas, 2010). PDK1 has been reported as a ‘master kinase’ due to its ability to activate numerous other members of the AGC kinase family, such as Akt, S6K, RSK, and PKC, by phosphorylating their activation loop (T-loop) (Pearce et al., 2010). This function highlights the significant role of PDK1 and emphasizes its capacity as a central player in regulating AGC kinases. Aberrant activation of downstream effectors of PDK1 and alterations in PDK1 itself have been reported in the context of cancer, which can lead to a poor prognosis and shorter overall survival (Gagliardi et al., 2018). As a result, PDK1 has emerged as a highly promising target for cancer therapy. Encouragingly, the use of PDK1 inhibitors in cancer treatment has shown significant efficacy in regulating the growth and survival of cancer cells (Emmanouilidi and Falasca, 2017). Nonetheless, further investigations are required to explore the outcomes of PDK1 inhibition in the tumor microenvironment, including investigations in vascular endothelial cells.

Previously, we showed that blockade of the PH domain of PDK1 using a small cell-penetrating peptide suppressed phosphorylation levels of PDK1 and Akt, which resulted in a significant reduction in the viability and tube-forming ability of human umbilical vein endothelial cells (HUVECs), as well as a noticeable decrease in both pathological retinal angiogenesis and tumor vessel formation (Park et al., 2015). In the present study, we created a novel, non-peptide, small molecule that inhibits the PH domain of PDK1, named CU05-1189. The antiangiogenic properties of CU05-1189 were examined *in vitro* using HUVECs, and the underlying mechanisms through which it exerts its antiangiogenic activity were thoroughly explored. More importantly, we conducted *in vivo* studies using a Matrigel plug assay and xenograft mouse models, which further supported the compound’s antiangiogenic effects. This study is the first to provide a theoretical framework and guidance for the possible use of a novel PH domain inhibitor of PDK1, CU05-1189, as a treatment for angiogenesis-related diseases such as cancer.

## 2 Materials and methods

### 2.1 Chemicals and reagents

The synthesis and purification of CU05-1189 were carried out by Curacle (Seoul, Korea) (99.5% purity, high-performance liquid chromatography). A 30-mM stock solution of CU05-1189 was prepared in dimethyl sulfoxide (DMSO) (Sigma-Aldrich, MO, United States, cat# D2650), aliquoted, and stored at −20°C until use. Antibodies against p-PDK1 S241 (cat# 3438S), PDK1 (cat# 3062S), S6K (cat# 9202S), p-Akt T308 (cat# 2965S), p-Akt S473 (cat# 9271S), Akt (cat# 9272S), p-mTOR S2448 (cat# 2971S), mTOR (cat# 2972S), p-GSK3β S9 (cat# 9323S), and GSK3β (cat# 9315S) were purchased from Cell Signaling Technology (MA, United States). Anti-p-S6K T229 (cat# ab5231) and GAPDH (cat# ab9484) antibodies were supplied by Abcam (Cambridge, United Kingdom). β-actin (cat# sc-47778) and VE-cadherin (cat# sc-9989) antibodies were obtained from Santa Cruz Biotechnology (TX, United States).

### 2.2 Cell lines and cell culture

HUVECs (ScienCell, CA, United States, cat# 8000) were cultured in endothelial cell medium (ScienCell, cat# 1001) supplemented with 5% fetal bovine serum (FBS), 1% endothelial cell growth supplement, and 1% penicillin/streptomycin. Cells were routinely passaged by trypsinization at approximately 90% confluency, and passages 3–6 were utilized for experiments. Cells were incubated at 37°C under a 5% humidified 95%:5% (v/v) mixture of air and CO₂.

### 2.3 Ligand-protein docking

**FIGURE 1:**
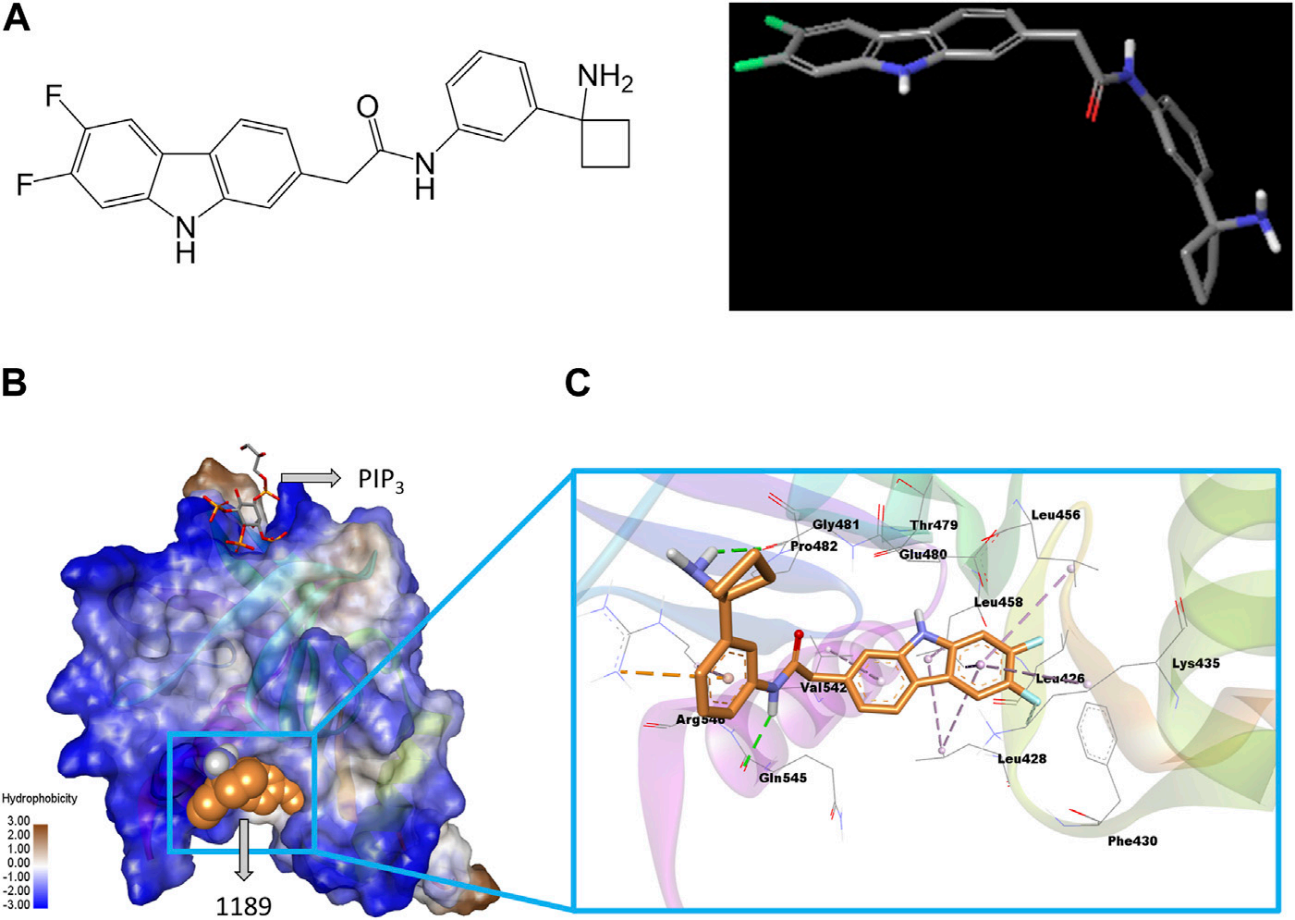
Structure of CU05-1189 and its predicted binding interactions with the PH domain of PDK1. **(A)** The 2D (left) and 3D (right) structure of compound 1189. In the 3D geometry-optimized 1189 structure, carbon, fluorine, nitrogen, and oxygen atoms are shown in grey, green, blue, and red, respectively. **(B)** The PH domain of PDK1 is shown in a hydrophobicity surface model. Blue and brown contours represent hydrophilic and hydrophobic surfaces, respectively. A co-crystal ligand (PIP_3_) is shown by sticks (carbon, grey; oxygen, red; sulfur, yellow), and the CU05-1189 binding conformation is shown by orange spheres in the model. **(C)** A close-up view of the CU05-1189 docked model is shown superimposed on 4-Å surrounding amino acid residues. The ligand (CU05-1189) is shown by sticks (carbon, orange; fluorine, cyan; nitrogen, blue; oxygen, red; and polar hydrogen, white), and interacting residues are shown by lines in the model. The protein structure is transparent in the secondary structure model. (Figure prepared using Discovery Studio Visualizer).

A geometry-optimized ligand (CU05-1189) structure was utilized for the docking experiment (Figure 1A). The Sitemap module of Maestro (Schrodinger Release 2022-3: Maestro, Schrodinger, LLC, NY, United States, 2021) was used to characterize the potential binding sites available on the PH domain of PDK1 and to assess the druggability of the sites (Halgren, 2009). The Sitemap program was used to analyze the whole protein to identify binding sites for which the size, functionality, and extent of pocket depth met the specifications. Then, the SiteScore algorithm ranked the possible binding sites and filtered out non-relevant sites based on the propensity of the site for ligand binding. The Sitemap module generated a map of hydrophobic (yellow), hydrogen bond donor (blue), and hydrogen bond acceptor (red) groups in the identified sites. Among the topmost binding sites identified by the Sitemap module, site 1 was chosen due to its larger volume and was used for the docking calculation of CU05-1189 (Supplementary Figure S2A). Site 1 was composed of the Leu426, Leu428, Phe430, Leu478, Glu480, Pro482, and Gln545 residues. A grid box center was assigned to these residues to perform induced-fit docking. A grid point was used to represent the most accurate description of the shape and different field properties that were utilized to score ligand poses. A density functional theory-optimized CU05-1189 structure was used for the docking calculations. Conformational sampling of ligand rings was performed using an energy window of 2.5 kcal/mol. A default glide docking option was used. Docked solutions of the ligand were refined using prime refinement, where side chains within 5 Å of ligand poses were optimized to obtain correct dihedral angles and minimized to remove clashes. The structures within 30 kcal/mol of the best-docked conformation were redocked using an extra-precision (XP) glide docking algorithm (Friesner et al., 2006), and 20 ligand-protein docked solutions were produced. An OPLS4 force field was used to parameterize ligands. Visual inspection of each pose was carried out to select a pose that fits well inside the putative binding pocket and had a more negative glide docking score.

### 2.4 Cell proliferation assay

Cell proliferation was analyzed using a 3-(4, 5-dimethyl-2-thiazolyl)-2, 5-diphenyl-2H-tetrazolium bromide (MTT) assay (Sigma-Aldrich, cat# M5655). Briefly, HUVECs (approximately 3 × 10^4^ cells/well) were seeded onto 2% gelatin-coated, 24-well tissue culture plates and incubated overnight for attachment. Next, the cultivation medium was replaced with Medium 199 (Corning, NY, United States, ref# 10-060-CV) supplemented with 1% FBS (HyClone, United States, cat# SH30919.03) in the presence or absence of VEGF (BioLegend, CA, United States, cat# 718302). Then, HUVECs were treated with 0.1% DMSO, which served as a vehicle control, or various concentrations of CU05-1189. After the indicated incubation time, the medium was removed, and MTT (0.1 mg/mL) was added for 4 h. The residual MTT was carefully removed, and DMSO:ethanol (1:1) was used to dissolve the formazan crystals. The absorbance was measured at a wavelength of 560 nm using a FLUOstar Omega microplate reader (BMG Labtech, Ortenberg, Germany).

### 2.5 Lactate dehydrogenase (LDH) assay

LDH release was measured using a CytoTox96^®^ Non-Radio Cytotoxicity Assay (Promega, WI, United States, ref# G1781) according to the manufacturer’s instructions. In brief, HUVECs were plated in gelatinized 24-well tissue culture plates and incubated overnight for attachment. After incubation with different concentrations of CU05-1189 for 24 h, the cell supernatants were collected from each well and analyzed to measure LDH release. The absorbance was determined at 490 nm using a microplate reader.

### 2.6 Wound-scratch migration assay

Cell migration was quantified using an *in vitro* wound-healing assay. HUVECs were seeded in 2% gelatin-coated, 12-well tissue culture plates and allowed to grow into a confluent monolayer. Next, the cells were serum-starved and scraped away using a sterile 1-mL pipette tip to create wounds. After washing the cells twice to remove detached cells, fresh Medium 199 containing 1% FBS with or without VEGF (30 ng/mL) was added to each well, followed by treatment with different concentrations of CU05-1189. Images were obtained at 0 and 16 h of incubation using an optical microscope. Wound closure was calculated using the formula (width_0h_–width_16h_)/width_0h_×100(%) and was expressed as a percentage of that in the vehicle (100%) without VEGF group.

### 2.7 Transwell invasion assay

An invasion assay was performed using 24-well Transwell permeable supports with 8.0-μm pores (Corning, cat# 3422). Aliquots of serum-starved HUVECs were resuspended in Medium 199 containing 1% FBS and seeded with a density of 1 × 10^5^ cells/well into the upper chambers of the Transwells, which were pre-coated with 0.1% gelatin. The lower chambers were filled with Medium 199 containing 1% FBS with or without VEGF (30 ng/mL) together with different concentrations of CU05-1189. After 4 h of incubation at 37°C, non-invasive cells on the upper membrane were removed by wiping with cotton swabs. Next, the inserts were fixed with methanol and stained with hematoxylin and eosin. Images were obtained with an optical microscope, and the invasion rate was expressed as a percentage of that of the vehicle without VEGF group (100%).

### 2.8 Capillary tube formation assay

A 24-well tissue culture plate was pre-coated with 250 μL of growth factor-reduced Matrigel Matrix Basement Membrane (Corning, cat# 354230). After solidification at 37°C for 30 min, serum-starved HUVECs (1.2 × 10^5^ cells/well) were seeded in 1% FBS-supplemented Medium 199 with or without VEGF (30 ng/mL) containing various concentrations of CU05-1189. After 6 h, the cells were photographed with an optical microscope. The tube length was expressed as a percentage of that of the vehicle without VEGF group (100%).

### 2.9 Western blot analysis

HUVECs were harvested with radioimmunoprecipitation assay buffer (100 mM Tris-Cl, 5 mM EDTA, 50 mM NaCl, 50 mM β-glycero-phosphate, 50 mM NaF, 0.1 mM Na_3_VO_4_, 0.5% NP-40, 1% Triton X-100, 0.5% sodium deoxycholate) containing a proteinase inhibitor cocktail (ThermoFisher Scientific, MA, United States, ref# A32961). Protein concentrations were determined using a SMART BCA Protein Assay kit (iNtRON, Gyeonggi-do, Korea, cat# 21071). Equivalent amounts of protein lysates were subjected to sodium dodecyl sulfate-polyacrylamide gel electrophoresis and transferred onto polyvinylidene fluoride membranes. The membranes were blocked with 3% bovine serum albumin in 0.1% TBS-T and immunolabeled with each specific primary antibody. Protein bands were visualized by an enhanced chemiluminescence method using enhanced chemiluminescence kits.

### 2.10 Subcellular fractionation

Cytoplasmic and membrane fractions were prepared as described previously with minor modifications (Zhang et al., 2017a). In brief, a buffer containing 50 mM Tris-Cl (pH 7.4), 100 mM NaCl, 0.01% digitonin, and proteinase inhibitor cocktail (ThermoFisher Scientific, ref# A32961) was used to harvest the cells and collect cytosolic supernatants. Then, the pellets were suspended in a buffer containing 50 mM Tris-Cl (pH 7.4), 100 mM NaCl, 2% Triton X-100, and proteinase inhibitor cocktail to perform membrane fractionation. After 30 min of incubation on ice, membrane fractionation was achieved by centrifugation for 5 min at 4°C. The concentration of each fraction was measured using a SMART BCA Protein Assay kit (iNtRON, cat# 21071), and the fractions were subjected to Western blotting.

### 2.11 Matrigel plug assay

Male C57BL/6 mice (5–6 weeks old) were supplied by DBL (Seoul, Korea), maintained in a semi-specific pathogen-free room at an adequate temperature (23°C) and humidity (60%) with a 12/12-h light/dark cycle, and subjected to a Matrigel plug assay as described previously with minor modifications (Park et al., 2015). Matrigel Matrix Basement Membrane (Corning, cat# 354230, 500 μL/plug) containing heparin (Sigma-Aldrich, cat# H3149, 20U) and recombinant mouse VEGF (BioLegend, cat# 718402, 200 ng) with various concentrations of CU05-1189 (10 or 50 μM) was subcutaneously injected near the axillary fossa of mice. Matrigel without VEGF served as a control. After 7 days of implantation, the Matrigel plugs were harvested and weighed. The hemoglobin content was quantified using a QuantiChrom™ Hemoglobin Assay Kit (BioAssay Systems, CA, United States, cat# DIHB-250) according to the manufacturer’s instructions. For immunofluorescence analysis, the Matrigel plugs were fixed in 4% paraformaldehyde and embedded with OCT compound (Sakura Finetek, CA, United States, ref# 4583). The microvessel density was determined using CD31 staining (R&D systems, MN, United States, cat# AF3628, 1:200).

### 2.12 *In vivo* subcutaneous tumor growth xenografts

Male BALB/c nude mice (5–6 weeks old) were purchased from DBL and housed in a semi-specific pathogen-free room at an adequate temperature (23°C) and humidity (60%) with a 12/12-h light/dark cycle. The animals were provided with free access to water and food. All animals were allowed to acclimatize to their new environment for 1 week prior to use. Experiments were conducted under protocols and conditions approved by the Institutional Animal Care and Use Committee of Yonsei University. Human non-small cell lung cancer cells (A549, 3 × 10^6^ cells) were suspended in 100 μL of Hanks’ balanced salt solution (Gibco, NY, United States, ref# 14025-092) and were subcutaneously inoculated into each mouse. When the tumors reached approximately 100 mm^3^, mice were randomly divided into four groups (n = 7–9/group). CU05-1189 was dissolved in 5% Tween-80 in distilled water, and 5% Tween-80 without the compound served as the vehicle. The vehicle and 12.5, 25, and 50 mg/kg CU05-1189 were each administered orally for 5 consecutive days followed by resting for 2 sequential days, and this cycle was repeated for 31 days. The body weight and tumor size of each mouse were recorded three times per week. The formula used to calculate the tumor volume was as follows: (length x width^2^) x 0.523. After sacrificing the mice on day 31, the tumors were dissected, photographed, weighed, and used for histological analyses.

### 2.13 Immunofluorescence and immunohistochemical analyses

Tumors were fixed in 4% paraformaldehyde overnight at 4°C and processed to produce either frozen or paraffin sections. OCT-embedded tissues were sectioned, stained with an anti-CD31 primary antibody (R&D systems, cat# AF3628, 1:200), and treated with 1 μg/mL of 4′,6-diamidino-2-phenylindole (DAPI) (Sigma-Aldrich, cat# 9542) for nuclear staining. After mounting, the slices were photographed using a confocal microscope (LSM 880, Carl Zeiss, Jena, Germany).

For paraffin section immunohistochemistry, the slides were incubated with anti-Ki67 (BD Biosciences, CA, United States, cat# 550609, 1:200) or anti-cleaved caspase-3 (Cell Signaling Technology, cat# 9661L, 1:400) primary antibodies. The cell nuclei were counterstained with hematoxylin (Merck, Darmstadt, Germany, CI# 75290), and images were captured using an Eclipse microscope (Nikon, Tokyo, Japan).

### 2.14 Statistical analysis

Statistical analysis was performed using GraphPad Prism 8.4.3 (GraphPad Software, CA, United States). The results are presented as the mean ± SEM. Data were statistically analyzed using one-way or two-way analysis of variance, followed by Tukey’s test. A *p*-value less than 0.05 was considered to indicate statistical significance.

## 3 Results

### 3.1 Identification of CU05-1189 and its interaction with the PH domain of PDK1

Our previous research demonstrated that blocking the PH domain of PDK1 could potentially have a therapeutic effect in treating pathological conditions by preventing angiogenesis (Park et al., 2015). Hence, we attempted to create a novel antiangiogenic agent by developing an inhibitor that targets the PH domain of PDK1 (Supplementary Figure S1A). In brief, we used a virtual screening method to examine a library consisting of approximately 300,000 compounds. We found one structure that could potentially bind to the PH domain of PDK1, and we created more than 500 agents through chemical synthesis based on this compound. HUVECs, which are endothelial cells frequently used to imitate tumor vasculature (Richardson et al., 2010), were selected for the *in vitro* investigations. We examined the effect of the synthesized compounds on VEGF-induced HUVEC proliferation, and some compounds were additionally evaluated for cytotoxicity and their ability to inhibit Akt phosphorylation. After taking into account various factors such as solubility and pharmacokinetics, several compounds were once more selected and tested to assess their *in vivo* antitumor activity using a xenograft mouse model. Among these, CU05-1189 was chosen for further studies (Figure 1A), and its detailed binding mechanism at the PH domain of PDK1 was investigated via a molecular docking simulation.

The sitemap indicated a putative ligand binding site of the PDK1 PH domain along with a co-crystal (PIP_3_) binding site (Supplementary Figure S2A). A representative docked pose was chosen based on the highly negative glide docking score (−7.21 kcal/mol) (Figure 1B). A two-dimensional (2D) representation of the docked model of CU05-1189 inside the PDK1 PH domain with different color contours for each interaction is illustrated in Supplementary Figure S2B. A 3D docked model showed that the central core (tricyclic) structure of CU05-1189 fit properly inside the hydrophobic pocket lined by the Leu426, Leu428, Phe430, Leu456, Leu458, Leu478, Pro482, and Val542 residues. The acetamide ‘NH’ of the ligand formed a hydrogen bond interaction with the main chain carbonyl ‘O’ of Gln545, whereas the amino-cyclobutyl moiety paired with Gly481 through a hydrogen bond interaction (green dash line). A phenyl ring formed a pi-cation interaction with Arg546 (orange dashed line). The carbazole ring scaffold formed pi-alkyl interactions with the Leu428, Lys435, Leu456, Leu458, and Val542 residues (lavender dashed lines). The 6,7-difluoro atoms on the carbazole ring were pointed toward Phe430, and 6-fluoro formed a van der Waals interaction with the side chain of Lys435. An ‘NH’ group of the carbazole ring was pointed toward the main chain carbonyl ‘O’ of Thr479. The hydrophobic part of the ligand docked inside the hydrophobic crevices, and the terminal hydrophilic part paired via a hydrogen bond with the hydrophilic residues (Figure 1C). These results suggested that CU05-1189 is an allosteric inhibitor of PDK1 that may interact with the PH domain of the kinase.

### 3.2 CU05-1189 suppresses VEGF-mediated cell proliferation of HUVECs *in vitro*

**FIGURE 2:**
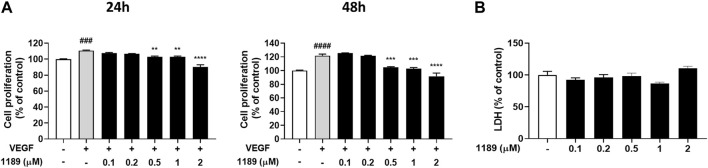
CU05-1189 inhibited VEGF-induced proliferation of HUVECs. **(A)** HUVECs were treated with various concentrations of CU05-1189 with or without 30 ng/mL VEGF for 24 h (left) or 48 h (right), and cell proliferation was measured using an MTT assay. Representative data are indicated as means ± SEM, *n* = 3. (^###^ *p* < 0.001, ^####^ *p* < 0.0001, VEGF-treated group vs. control group; **p < 0.01, ***p < 0.001, ****p < 0.0001, VEGF and CU05-1189-treated group vs. VEGF-treated group). **(B)** LDH release from HUVECs was measured after treatment with various concentrations of CU05-1189 for 24 h. Data are represented as means ± SEM, *n* = 3.

Because growth factors, specifically VEGF, play a key role in initiating angiogenesis (Hicklin and Ellis, 2005; Niu and Chen, 2010), and endothelial cell proliferation is essential for the development of new blood vessels within tumors (Jiang et al., 2020), we examined whether CU05-1189 could inhibit VEGF-mediated proliferation of HUVECs. The MTT assay results showed that VEGF-induced HUVEC proliferation was markedly attenuated after CU05-1189 treatment in a dose- and time-dependent manner (Figure 2A). To further determine whether the decrease in proliferation caused by CU05-1189 was a result of its toxic effects on HUVECs, an LDH release assay was conducted. As shown in Figure 2B, CU05-1189 treatments ranging from 0.1 to 2 μM caused no apparent toxicity to HUVECs. Based on these findings, we selected 2 μM CU05-1189 as a maximum concentration for further *in vitro* experiments. Taken together, our data suggest that CU05-1189 is a potent inhibitor of VEGF-induced HUVEC proliferation *in vitro*.

### 3.3 CU05-1189 blocks VEGF-induced endothelial cell migration, invasion, and tube formation

**FIGURE 3:**
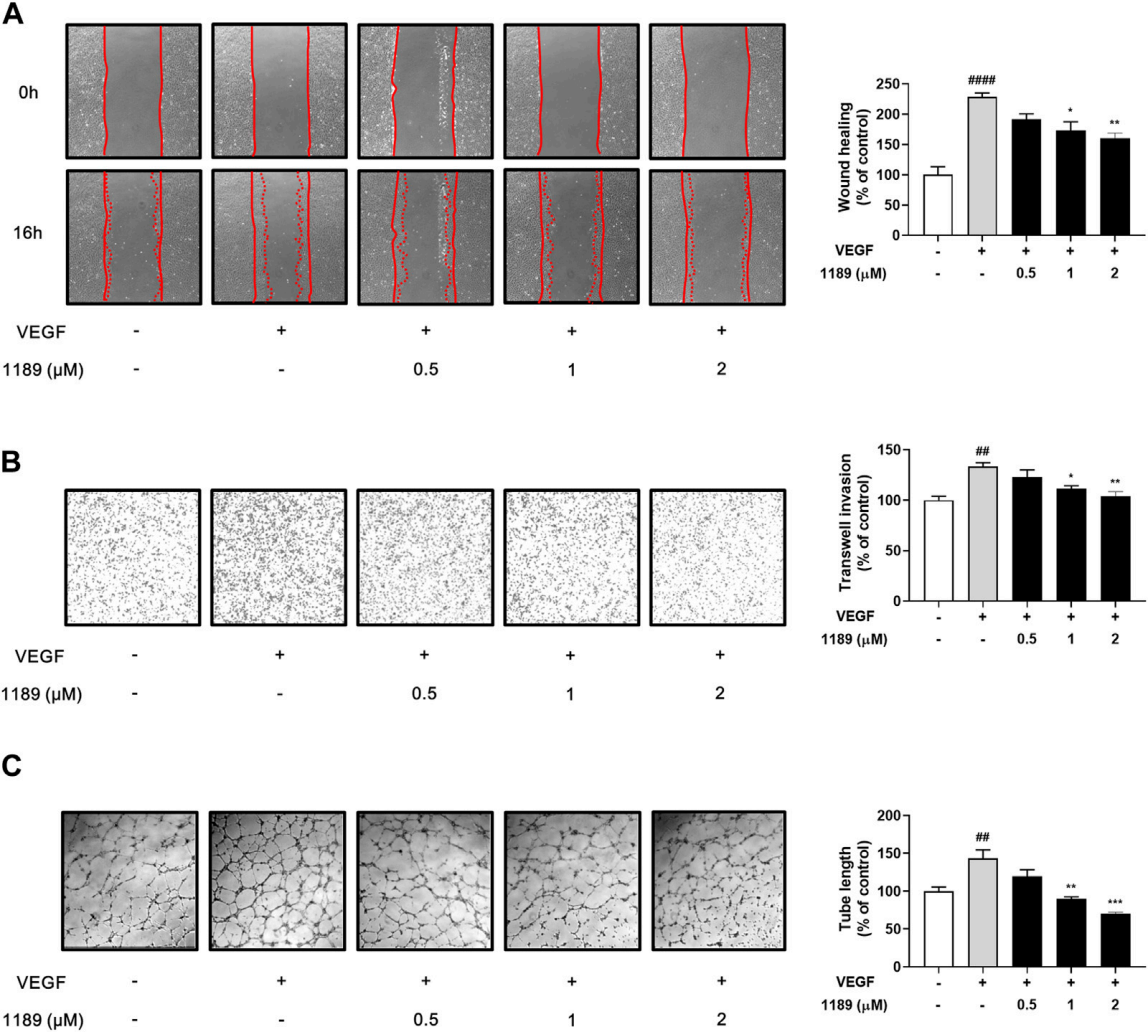
CU05-1189 suppressed VEGF-induced migration, invasion, and tube formation by HUVECs. **(A)** Representative images and quantitative data of HUVEC migration after treatment with CU05-1189 in the absence or presence of VEGF. The wound area was photographed at 0 and 16 h of incubation (magnification, ×40). **(B)** Representative images and quantitative data of HUVEC invasion after exposure to CU05-1189 in the absence or presence of VEGF. HUVEC invasion of the bottom of a 0.1% gelatin pre-coated Transwell was photographed at 4 h of incubation (magnification, ×40). **(C)** Representative images and quantitative data of tube formation by HUVECs after exposure to CU05-1189 in the absence or presence of VEGF. The tube formation images were obtained at 6 h of incubation (magnification, ×40). All data are represented as means ± SEM, *n* = 3 (^##^ *p* < 0.01, ^####^ *p* < 0.0001, VEGF-treated group vs. control group; *p < 0.05, **p < 0.01, ***p < 0.001, VEGF and CU05-1189-treated group vs. VEGF-treated group).

Vascular endothelial cell migration is a key process in angiogenesis (Lamalice et al., 2007; Norton and Popel, 2016). We thus investigated the potential effect of CU05-1189 on the chemotactic motility of HUVECs using a wound-healing assay. As shown in Figure 3A, the gap areas between the sides of scratches gradually increased when HUVECs were treated with increasing CU05-1189 concentrations. This finding indicates that CU05-1189 can inhibit VEGF-induced migration of HUVECs in a concentration-dependent manner. Next, the effects of CU05-1189 on vertical invasion of HUVECs were investigated using a Transwell invasion assay. The results confirmed that CU05-1189 dose-dependently inhibited HUVEC invasion in the presence of VEGF (Figure 3B). Moreover, maximum inhibition of endothelial cell invasion was observed with 2 μM CU05-1189, which was similar to that of the VEGF-absent control. We then conducted a 2D Matrigel tube formation assay to further assess the antiangiogenic effects of CU05-1189. The length of tubular structures formed by VEGF was decreased in a dose-dependent manner after incubation with 0.5–2 μM CU05-1189 (Figure 3C). These data indicate that CU05-1189 suppresses VEGF-triggered angiogenesis *in vitro* by inhibiting the migration, invasion, and tube formation of endothelial cells.

### 3.4 CU05-1189 inhibits the PDK1/Akt signaling pathway by suppressing membrane localization of PDK1

**FIGURE 4:**
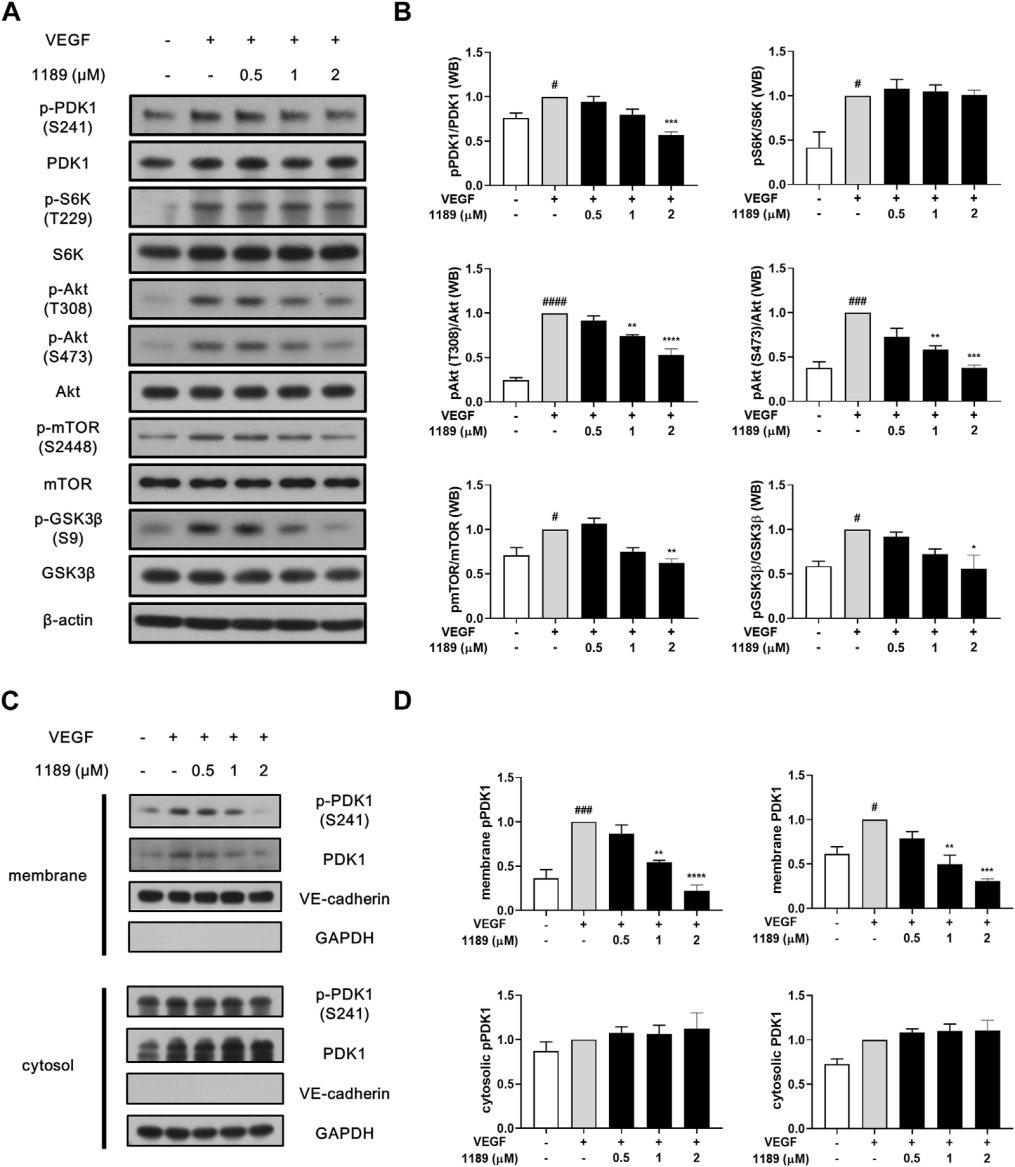
CU05-1189 inhibited the PDK1/Akt signaling axis by suppressing membrane localization of PDK1. **(A)** CU05-1189 inhibited phosphorylation of PDK1 and its major downstream effector, Akt, at the plasma membrane without affecting its cytosolic substrate, S6K, in HUVECs. Serum-starved HUVECs were pre-treated with various concentrations of CU05-1189 for 1 h before exposure to VEGF (30 ng/mL) for 10 min. Phosphorylation of PDK1, S6K, Akt, and the downstream cascade of Akt (mTOR and GSK3β) was examined by Western blotting. **(B)** Quantification of the grayscale ratio between each phosphorylated protein and the total protein. Data are represented as means ± SEM, *n* = 3 (#*p* < 0.05, ^###^ *p* < 0.001, ^####^ *p* < 0.0001, VEGF-treated group vs. control group; *p < 0.05, **p < 0.01, ***p < 0.001, ****p < 0.0001, VEGF and CU05-1189-treated group vs. VEGF-treated group). **(C)** Membrane and cytosolic fractions were prepared and immunoblotted for the indicated proteins. HUVECs were pre-treated with various concentrations of CU05-1189 for 1 h before exposure to VEGF (30 ng/mL) for 10 min. **(D)** Quantification of western blots in **(C)**. Data are presented as means ± SEM, n = 3 (^#^ *p* < 0.05, ^###^ *p* < 0.001, VEGF-treated group vs. control group; **p < 0.01, ***p < 0.001, ****p < 0.0001, VEGF and CU05-1189-treated group vs. VEGF-treated group).

Western blot analysis was performed to elucidate the molecular mechanism underlying the antiangiogenic effects of CU05-1189. The addition of exogenous VEGF potently induced phosphorylation of VEGF receptor 2, the main receptor of VEGF, and its downstream signaling molecules, including PI3K, FAK, ERK, and p38. However, CU05-1189 did not inhibit VEGF-induced phosphorylation of the aforementioned molecules (Supplementary Figure S3A). In contrast, CU05-1189 treatment effectively suppressed phosphorylation of PDK1 and Akt, which is the key downstream component of PDK1 (Hu et al., 2017). Moreover, the VEGF-mediated phosphorylation levels of two well-known downstream molecules of Akt, GSK3β and mTOR, exhibited concentration-dependent decreases after incubation with CU05-1189 (Figures 4A, B). These data imply that the antiangiogenic capability of CU05-1189 is achieved by selectively inhibiting the PDK1/Akt pathway.

Interestingly, the VEGF-stimulated phosphorylation of S6K, another substrate of PDK1, was not affected by CU05-1189 (Figures 4A, B). To determine how the PDK1-mediated Akt pathway is specifically regulated by CU05-1189, we conducted a subcellular fractionation analysis to define the cellular localization of PDK1. The results revealed that translocation of PDK1 to the plasma membrane upon VEGF stimulation was largely diminished by CU05-1189 in a dose-dependent manner, but this result was not observed in the cytosolic fraction. Additionally, comparable outcomes were observed in the localization of PDK1 that had been phosphorylated at S241 (Figures 4C, D). These results indicate that CU05-1189 may suppress localization of PDK1 to the plasma membrane, thus exceptionally inhibiting Akt activation.

### 3.5 CU05-1189 exerts antiangiogenic effects in matrigel plugs *in vivo*

**FIGURE 5:**
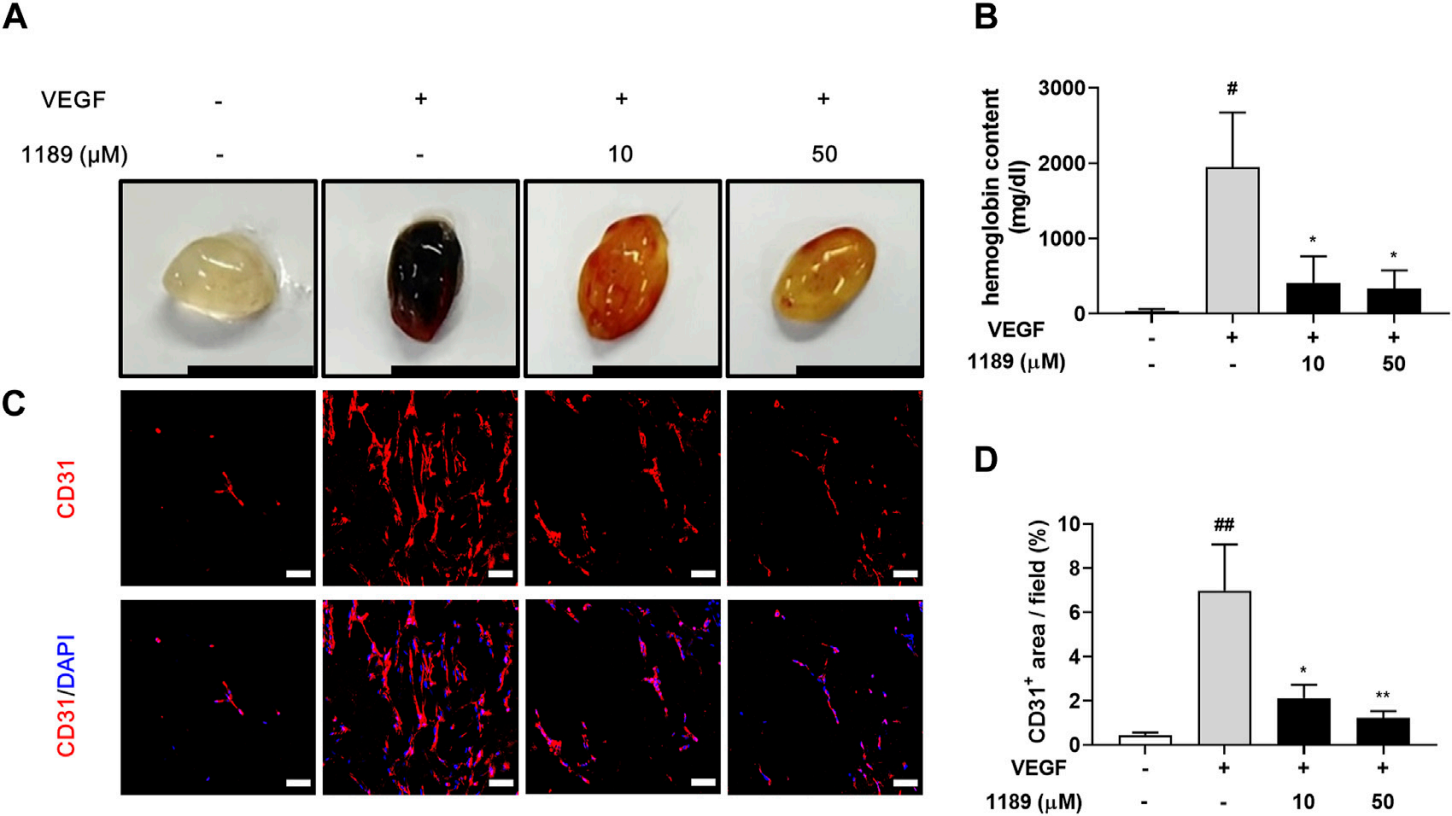
CU05-1189 blocked VEGF-induced angiogenesis in the murine Matrigel plug assay. **(A)** Representative photographs of Matrigel plugs excised from mice treated with different concentrations of CU05-1189 in the presence or absence of VEGF (scale bar: 1 cm). **(B)** Quantified hemoglobin content normalized to the weight of Matrigel plugs. Each value is presented as the mean ± SEM, *n* = 5–7. **(C)** Frozen sections of the Matrigel plugs were stained with an anti-CD31 antibody (red) and DAPI (blue). **(D)** The percentage of the CD31-positive area is presented as the mean ± SEM, *n* = 5 (^#^ *p* < 0.05, ^##^ *p* < 0.01, VEGF-treated group vs. control group; *p < 0.05, **p < 0.01, VEGF and CU05-1189-treated group vs. VEGF-treated group).

We next performed a Matrigel plug assay to further investigate whether CU05-1189 could inhibit angiogenesis *in vivo*. The formed plugs infused with VEGF appeared red, indicating abundant infiltration of the vasculature within the plugs. In contrast, the colors of Matrigel plugs containing both VEGF and CU05-1189 were much lighter than the color of the plugs from the VEGF-treated group (Figure 5A). We then quantified the level of angiogenesis by measuring the hemoglobin concentrations in the Matrigel plugs. A histogram shows that CU05-1189 markedly reduced neovascularization within the plugs compared with that in the VEGF-treated group (Figure 5B). Because CD31 is present on most capillaries and is widely used as an endothelial marker to quantify angiogenesis (Wang et al., 2008), we used an anti-CD31 antibody to further clarify the number of functional vessels in the Matrigel plugs. CU05-1189 markedly reduced VEGF-induced vessel formation in a dose-dependent manner (Figures 5C, D). These data indicate that CU05-1189 significantly inhibits angiogenesis *in vivo*.

### 3.6 Orally active CU05-1189 inhibits tumor angiogenesis and growth in an A549 xenograft mouse model

**FIGURE 6:**
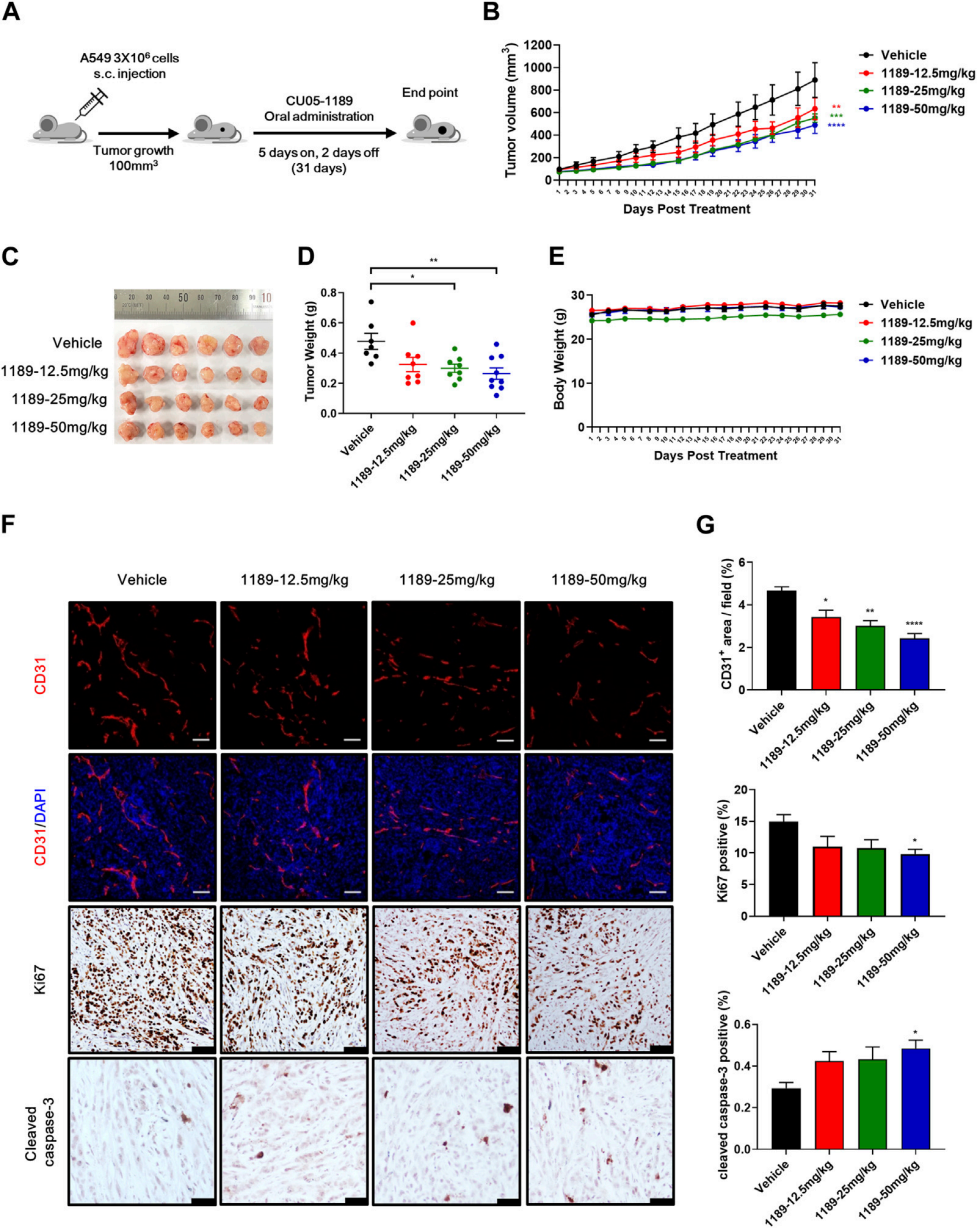
CU05-1189 inhibited tumor angiogenesis and growth in an A549 xenograft mouse model. **(A)** Experimental scheme. When the tumor volume reached approximately 100 mm^3^, the mice were treated with vehicle or the indicated dose of CU05-1189 by oral administration for 5 consecutive days followed by resting for 2 sequential days, which was repeated for 31 days. **(B)** *In vivo* growth curve of A549 xenograft tumors of mice treated with vehicle and the indicated doses of CU05-1189. **(C)** Photographs of resected tumors on day 31. **(D)** Tumor weight on the last day of the experiment. **(E)** Mouse body weight. **(F)** A549 tumor tissues were stained with anti-CD31 (red staining), anti-Ki67 (brown staining), and anti-cleaved caspase-3 (brown staining) antibodies (scale bar: 100 μm, except for cleaved caspase-3: 50 μm). **(G)** Quantified results of **(F)**. Each value is presented as the mean ± SEM, n = 7–9 in A–E and *n* = 5–6 in F, G (*p < 0.05, **p < 0.01, ***p < 0.001, ****p < 0.0001, CU05-1189-treated group vs. vehicle-treated group).

The antiangiogenic activities of CU05-1189 in VEGF-induced endothelial cells and Matrigel plug models prompted us to examine whether CU05-1189 could inhibit tumor growth by suppressing angiogenesis. Considering the favorable bioavailability (68%) of CU05-1189 indicated in our pharmacokinetic studies (Supplementary Table S1), we selected oral administration as the preferred method of treatment. When tumors reached a volume of approximately 100 mm^3^, CU05-1189 was added at doses of 12.5, 25, and 50 mg/kg for 5 consecutive days followed by resting for 2 sequential days, and the cycle was repeated for 31 days (Figure 6A). In the vehicle-treated group, tumors rapidly increased in size after 31 days, while in all CU05-1189-treated groups, tumor growth was considerably slower. This finding indicates that CU05-1189 significantly hindered tumor growth in a dose-dependent manner. On the last day of the test, the average tumor volumes in groups treated with 12.5, 25, and 50 mg/kg CU05-1189 were 632.74 ± 97.36, 549.94 ± 38.15, and 487.90 ± 73.72 mm^3^, respectively, whereas the tumor volume of the control group was 888.98 ± 154.01 mm^3^ (Figures 6B, C). Correspondingly, the average tumor weights at these doses were 0.33 ± 0.05, 0.3 ± 0.03, and 0.26 ± 0.04 g, respectively, while the tumor weight of the vehicle-treated group was 0.48 ± 0.05 g (Figure 6D). However, the mice did not experience any significant loss of body weight at these doses (Figure 6E). Furthermore, the highest dose of CU05-1189 at 50 mg/kg did not elicit any significant changes in the relative organ weights (organ to body weight ratio), as evidenced in Supplementary Figure S4A, which serves as an indicator of organ toxicity (Lazic et al., 2020). There were no obvious differences in the levels of biochemical parameters (GOT, GPT and blood glucose) between the group treated with the vehicle and the one treated with CU05-1189 at 50 mg/kg (Supplementary Figures S4B, C). These findings further support that CU05-1189 can hinder tumor growth without causing significant side effects, such as hepatic toxicity or hyperglycemia.

To further investigate whether CU05-1189 inhibited tumor angiogenesis, an immunohistochemical examination using an anti-CD31 antibody was conducted to stain A549 tumor tissue sections. Analysis of CD31-immunostained tissue revealed a significant reduction of microvessel density in tumors treated with CU05-1189 in a dose-dependent manner. Furthermore, CU05-1189 not only inhibited tumor cell proliferation but also increased the tumor apoptotic area, showing statistical significance at a dose of 50 mg/kg (Figures 6F, G). Collectively, these data from the A549 xenograft mouse model suggest that CU05-1189 may suppress tumor growth by inhibiting tumor angiogenesis.

## 4 Discussion

Targeting of angiogenesis has been considered an effective strategy for treating cancer due to the known promotion of tumor growth by excessive and uncontrolled angiogenesis. Our prior research proposed a new strategy to target angiogenesis, which involved blocking the PH domain of PDK1 and subsequent inhibition of the Akt pathway with a small cell-penetrating peptide, and demonstrated its potential therapeutic implications (Park et al., 2015). Based on these findings, we sought to develop and synthesize a novel orally-available small molecule. From our series of newly-synthesized compounds, we ultimately identified a compound called CU05-1189, which was verified to bind to the PH domain of PDK1 (Figures 1B, C). The antiangiogenic effects of this compound and the underlying mechanism were thoroughly investigated to demonstrate its potential as a novel antiangiogenic agent for therapeutic use.

Among many angiogenic stimulators, VEGF is a key molecule secreted by tumor cells during the angiogenic switch. Therefore, our first approach to test the antiangiogenic effect of CU05-1189 was to investigate whether the compound may inhibit major steps involved in VEGF-induced angiogenesis. Our results revealed that VEGF-stimulated proliferation, migration, invasion, and capillary-like tube formation of HUVECs were significantly suppressed by CU05-1189 in a dose-dependent manner (Figures 2A, 3A–C). Importantly, the antiangiogenic effects of CU05-1189 were not associated with any toxic effects, as evidenced by an LDH assay (Figure 2B).

The molecular docking simulation findings suggested that the primary molecular mechanism of the antiangiogenic activity of CU05-1189 was likely to be inhibition of the PDK1 signaling pathway. Correspondingly, our Western blot analysis provided evidence of specific inhibition of the PDK1/Akt signaling pathway (Figures 4A, B). CU05-1189 reduced Akt phosphorylation at T308, which is in the T-loop typically phosphorylated by PDK1. Additionally, CU05-1189 reduced phosphorylation at S473, which is in the H-motif necessary for full activation of Akt. This finding is consistent with our previous investigation (Park et al., 2015) and a study by other researchers using the PDK1 inhibitor 2-*O*-Bn-InsP_5_, which also inhibited both T308 and S473 phosphorylation in the SKOV-3 ovarian cancer cell line (Falasca et al., 2010). Moreover, CU05-1189 suppressed phosphorylation of GSK3β and mTOR, which are involved in cell growth and protein synthesis, respectively, upon activation of Akt. These findings indicate that the antiangiogenic effects of CU05-1189 may be attributed, in part, to its ability to inhibit phosphorylation of several proteins involved in the PDK1/Akt pathway.

PDK1, recognized as a prominent master kinase, unquestionably plays a crucial role in regulating numerous kinases within the AGC kinase family. Consequently, growth factor-induced activation of substrates such as Akt or S6K cannot occur in mammalian cells that lack PDK1 (Mora et al., 2005; Pinner and Sahai, 2008). However, it is noteworthy that CU05-1189 did not induce a decrease in S6K phosphorylation, unlike Akt. Akt, among all the PDK1 substrates characterized, is the only one containing the PH domain, which enables its interaction with PIP_3_ (Bayascas, 2010). Both Akt and PDK1 are recruited to the plasma membrane, where a permissive conformational change of Akt occurs, facilitating its phosphorylation at the T-loop by PDK1 (Calleja et al., 2007). However, the other substrates activated by PDK1, including S6K, lack a PH domain and undergo phosphorylation by PDK1 in the cytosol, rather than in the plasma membrane (Mora et al., 2004). Previous research suggests that the presence of a PDK1-interacting fragment (PIF) pocket in PDK1, which serves as a docking site for its substrates, allows phosphorylation of these substrates in the cytosol (Bayascas, 2010; Leroux et al., 2018). This distinctive mechanism of Akt activation by PDK1, compared with other PDK1 substrates, helped us identify the localization of PDK1 after CU05-1189 treatment. The subcellular fractionation results implied that the specific inhibition of the Akt pathway may be attributed to the suppression of PDK1 membrane localization by CU05-1189 after stimulation with VEGF. Furthermore, the specific site in the PH domain of PDK1 blocked by CU05-1189 might not interfere with the PIF pocket, as otherwise phosphorylation of S6K at its T-loop would also have been inhibited.

The results obtained from the *in vitro* experiments were further verified by the Matrigel plug model, which revealed dose-dependent inhibition of VEGF-induced neovascularization in response to CU05-1189. Moreover, oral administration of CU05-1189 to tumor xenograft mice resulted in a marked decrease in the area of blood vessels within the tumor, which was also accompanied by a significant reduction in tumor proliferation at a dose of 50 mg/kg. In addition, the decrease in tumor neovascularization appeared to trigger an increase in tumor apoptosis, which could be due to the lack of survival factors that are typically supplied by blood vessels (Brakenhielm et al., 2004). Nonetheless, the alterations observed in tumor proliferation and apoptosis were relatively minimal compared to the changes in vessel area, especially at doses of 12.5 and 25 mg/kg. One possible explanation could be a threshold phenomenon, a hypothesis proposing that a certain level of angiogenesis inhibition is necessary to achieve statistically significant tumor death (Svensson et al., 2007). However, marked retardation in tumor growth was observed without obvious toxicity, suggesting that CU05-1189 demonstrated its effectiveness against tumor growth by impeding angiogenesis.

Numerous studies have demonstrated the pivotal role of the PDK1/Akt pathway in promoting tumor formation and growth factor-mediated angiogenesis, thereby rendering the pathway an attractive target for therapeutic intervention (Karar and Maity, 2011; He et al., 2021). In the present study, we demonstrated that blocking of the PH domain of PDK1 by CU05-1189 may lead to inhibition of angiogenesis by suppressing the Akt signaling pathway. This finding is compatible with numerous studies that have explored the potential of targeting the Akt signaling pathway as a means of effectively suppressing angiogenesis. One such example focused on the drug perifosine, which is a well-known Akt inhibitor, and demonstrated its potential as a therapeutic agent against increased angiogenesis (Wang et al., 2012). Additionally, other studies have proposed the efficacy of inhibiting the Akt pathway in suppressing angiogenesis both *in vitro* and *in vivo* (Nakashio et al., 2002; Fokas et al., 2012; Kuger et al., 2013; Wang et al., 2019; Srivastava et al., 2020). Moreover, specific inhibition of the Akt signaling pathway has been suggested to be sufficient to trigger antiangiogenic effects (Cho et al., 2017), further supporting that our results are in line with those of previous reports.

Despite the demonstrated effectiveness of several small molecules as PDK1 and Akt inhibitors both *in vitro* and *in vivo*, only a few have progressed to clinical trials for cancer treatment purposes (Landel et al., 2020). Such examples include UCN-01, a nonselective PDK1 inhibitor, which has shown promise in combination therapy with irinotecan for treatment of metastatic triple-negative breast cancer (Wang et al., 2022). Additionally, the Akt inhibitors capivasertib (AZD5363) and ipatasertib (GDC-0068) are undergoing phase III clinical trials for cancer therapy (Landel et al., 2020; Hua et al., 2021). However, to date, development of many of these small molecules has been primarily focused on ATP-competitive inhibition of kinases (Peifer and Alessi, 2008; Medina, 2013). The selectivity of these inhibitors may be restricted due to the similarity of ATP binding sites across serine/threonine kinases (Li, 2007). Therefore, the development of allosteric inhibitors has become necessary. As a response, several allosteric Akt inhibitors, including ARQ092, TAS-117, and MK-2206, have been administered to patients with advanced solid tumors in early-phase trials (Martorana et al., 2021). Although MK-2206 was the only agent that underwent further investigation, particularly in breast cancer subtypes, it did not show sufficient effectiveness at tolerable doses (Konopleva et al., 2014; Tamura et al., 2015; Xing et al., 2019). There is ongoing potential for the development of Akt inhibitors with improved pharmacokinetic properties (Ma et al., 2022). Furthermore, ensuring a favorable safety profile remains a significant challenge in the development of Akt-blocking agents, as previous studies have underscored the potential for side effects, such as rash, nausea, fatigue, hepatotoxicity, and hyperglycemia, associated with PI3K/Akt pathway inhibitors (Liu et al., 2019; Yu et al., 2023).

The significance of our newly-synthesized compound, CU05-1189, resides first in the achievement of developing a novel agent based on our previous research, which was an innovative attempt. We have successfully demonstrated that the PH domain of PDK1 could be an additional target site to potently inhibit the Akt pathway, which is consistent with previous reports (Falasca et al., 2010; Medina, 2013). Second, CU05-1189 is an orally-available small molecule that may offer advantages such as improved pharmacokinetic properties, reduced cost, easier drug storage, and greater patient compliance (Zhong et al., 2021). Finally, CU05-1189 demonstrated the ability to reduce tumor growth at commonly tested doses of 12.5, 25, and 50 mg/kg without causing significant toxicity (Falasca et al., 2010). Given that severe toxicities can ultimately result in the discontinuation of clinical trials for new compounds, it is particularly encouraging that CU05-1189 displayed good tolerability and no signs of toxicity associated with previous inhibitors targeting the Akt pathway. This finding bodes well for the potential for further research and development of this compound. Overall, CU05-1189 appears to fulfill the criteria for an ideal antiangiogenic drug, offering convenient administration, affordability, and low toxicity (Zhang et al., 2017b). To the best of our knowledge, this study is the first to demonstrate the inhibition of angiogenesis by a small molecule that blocks the translocalization of PDK1 to the plasma membrane and subsequently blocks the Akt pathway by inhibiting the PH domain of PDK1. However, the exact mechanism responsible for the impeded translocation and phosphorylation of PDK1 necessitates additional investigation for further clarification. One possible explanation is that the PH domain of PDK1 is exceptionally extended (Bayascas, 2010), allowing for the possibility of allosteric targeting of the kinase. CU05-1189 might impede the interaction between the PH domain and membrane-bound molecules other than PIP_3_.

To summarize, we have successfully created a novel PH domain inhibitor of PDK1, called CU05-1189, by building upon our prior research and experimentally revealed its antiangiogenic effects using an *in vitro* HUVEC model. The potential mechanism may involve blockade of the PDK1/Akt signaling pathway, which is accomplished by preventing the translocation of PDK1 to the plasma membrane. More importantly, the compound inhibited tumor growth and reduced microvessel density *in vivo*, indicating that CU05-1189 appears to be a potent, orally active, antiangiogenic agent that is worthy of further investigation.

## Data Availability

The original contributions presented in the study are included in the article/Supplementary Material, further inquiries can be directed to the corresponding author.
