# Supplementary material for: A novel small molecule, CU05-1189, targeting the pleckstrin homology domain of PDK1 suppresses VEGF-mediated angiogenesis and tumor growth by blocking the Akt signaling pathway
Source: Front Pharmacol. 2023 Nov 16;14:1275749. doi: 10.3389/fphar.2023.1275749 (PMC10687218; doi:10.3389/fphar.2023.1275749)
Supplement: Supplementary file 1 [file DataSheet1.docx]

Supplementary Material

A Novel Small Molecule, CU05-1189, Targeting the Pleckstrin Homology Domain of PDK1 Suppresses VEGF-mediated Angiogenesis and Tumor Growth by Blocking the Akt Signaling Pathway

Jeongeun Park^1^, Haiying Zhang^2^, Hyun Jung Kwak^3^, Changdev Gorakshnath Gadhe^4^, Yeomyeong Kim^1^, Hyejeong Kim^1^, Minyoung Noh^1^, Dongyun Shin^5^, Sang-Jun Ha^1^, Young-Guen Kwon^1*^

*** Correspondence:** Young-Guen Kwon: ygkwon@yonsei.ac.kr

# Supplementary Methods

**PDK1-PH domain protein modeling**

Human PDK1 deposited in the UniProtKB database under the codename O15530 was used. The X-ray crystal structure of the human PDK1 PH domain was downloaded from the Protein Data Bank (https://www.rcsb.org/) (PDB code: 1W1G, resolution=1.45 Å). The X-ray structure was co-crystallized with diC4-phosphatidylinositol (3,4,5)-triphosphate. Schrodinger’s Maestro package (Schrodinger Release 2022-3: Maestro, Schrodinger, LLC, New York, NY, USA, 2021) was utilized to perform all computational modeling and docking experiments. The X-ray structure was imported into Maestro and prepared using a protein preparation wizard at a pH level of 7.4. During the protein preparation steps, missing residues and sidechains were reconstructed, hydrogen atoms were added, protonation states of the ionizable residues were adjusted, tautomeric forms for histidine residues were defined, and water molecules were deleted. The OPLS4 force field was used to parameterize and minimize the structure. The processed structure coordinates were saved for the subsequent modeling step.

**Ligand modeling**

A 2D structure of CU05-1189 was drawn using ChemDraw v20.1.1 (<https://www.perkinelmer.com>). A ligand structure (CU05-1189) was saved in a structure data file (.sdf) format and was imported into Maestro for further processing. The default ligand preparation process of the LigPrep module was used with a pH level of 7.4. Furthermore, ligand full-geometry optimization was performed with the Jaguar module. The density functional theory method was used for ligand optimization with a 6-31G** basis set and a B3LYP-D3 functional hybrid. The self-consistent field spin was treated automatically with the maximum grid density. The accuracy level was set to ultrafine with an initial guess of the atomic overlap. For the self-consistent field, the maximum iteration was set to 100 steps with default parameters chosen for convergence criteria. For the geometry optimization, the maximum number of steps was set to 1000 with XYZ-cartesian coordinate optimization. The Poisson Boltzmann finite element method was used for solvation, with water as a solvent. At the end of the simulation, the surfaces (MO, density, and potential), atomic electrostatic potential charges, and Mulliken populations were saved.

**Chemicals and reagents**

Antibodies against p-VEGF receptor 2, VEGF receptor 2, p-FAK, FAK, p-PI3K, PI3K, p-ERK, ERK, p-p38, and p38 were purchased from Cell Signaling Technology (MA, United States).

**In vivo toxicity determination**

Following the daily oral administration of the vehicle and CU05-1189 at a dose of 50mg/kg for a duration of 27 days in an A549 xenograft model, the relative organ weights were calculated at the time of sacrifice using the formula: relative organ weight = [organ weight (g) / body weight (g)] x 100. To analyze hepatic parameters in the serum, glutamic oxaloacetic transaminase (GOT) and glutamic pyruvate transaminase (GPT) activities were measured using commercially available kits (Asan Pharmaceutical Co., Seoul, Korea, cat#AM102-K, cat#AM103-K). The blood glucose level was determined by obtaining samples from the tail vein using a glucometer (Roche, Germany, Accu-Chek).

**Pharmacokinetic study**

A pharmacokinetic study was performed to assess the bioavailability of CU05-1189 in male Sprague-Dawley rats (8 weeks old). After drug administration, the collected blood samples were centrifuged at 13,000 rpm for 2 min to harvest plasma. For each plasma sample, a 10-μl aliquot was combined with 20 μl of ISTD (0.2 μg/ml carprofen in methanol) followed by addition of 100 μl of acetonitrile. The mixture was vortexed for 1 min and then centrifuged at 14,000 rpm for 5 min. Next, 5 μl of the resulting sample was used for liquid chromatography with tandem mass spectrometry (Shimadzu LC-MS/MS 8050). The pharmacokinetic parameters were analyzed using LabSolutions software. The oral bioavailability (*F*) was calculated according to the following equation: *F*=AUC_(0-_*_t_*_)_ (per oral, p.o.)/AUC_(0-∞)_ (intravenous, i.v.) x dose (i.v.)/dose (p.o.) x 100(%).

# Supplementary Figures and Tables

## Supplementary Figures


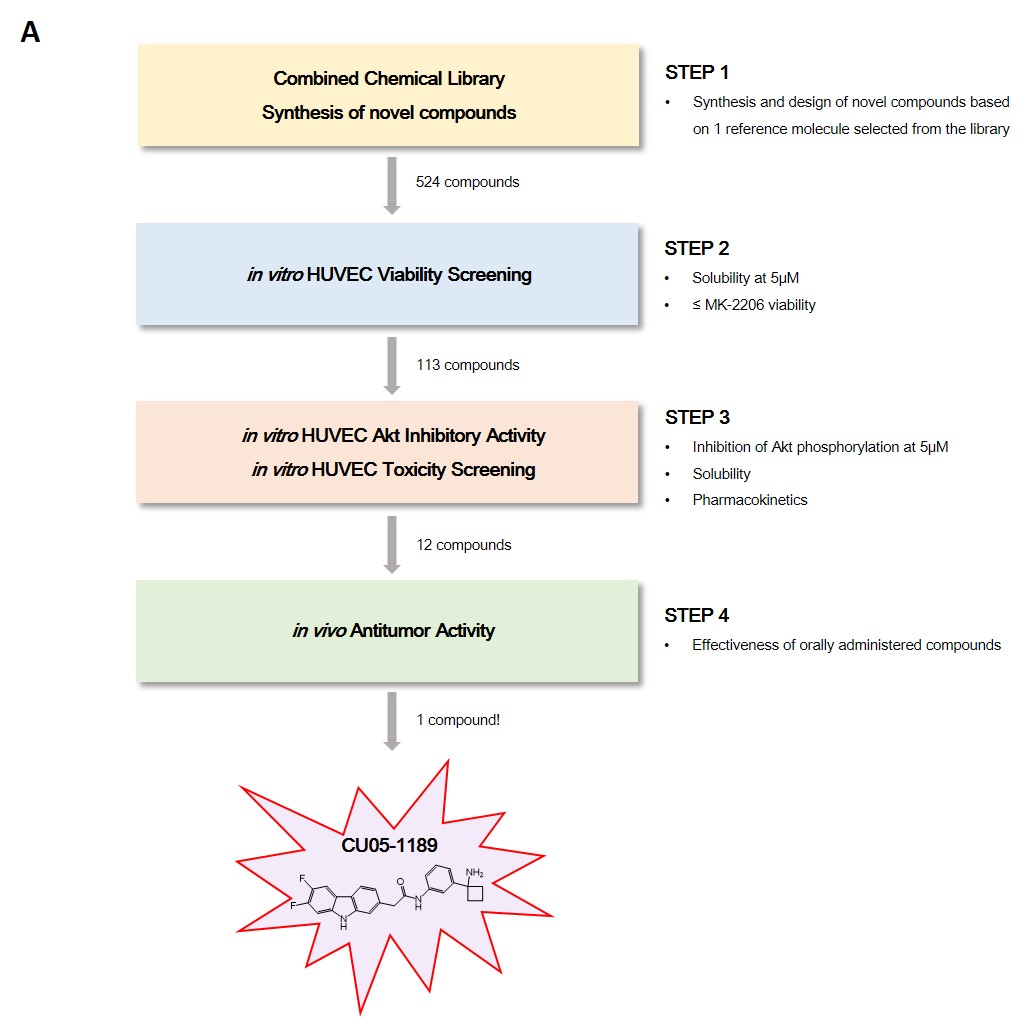


**Supplementary Figure 1.** **(A)** A brief schematic diagram of our screening procedure for selecting CU05-1189.


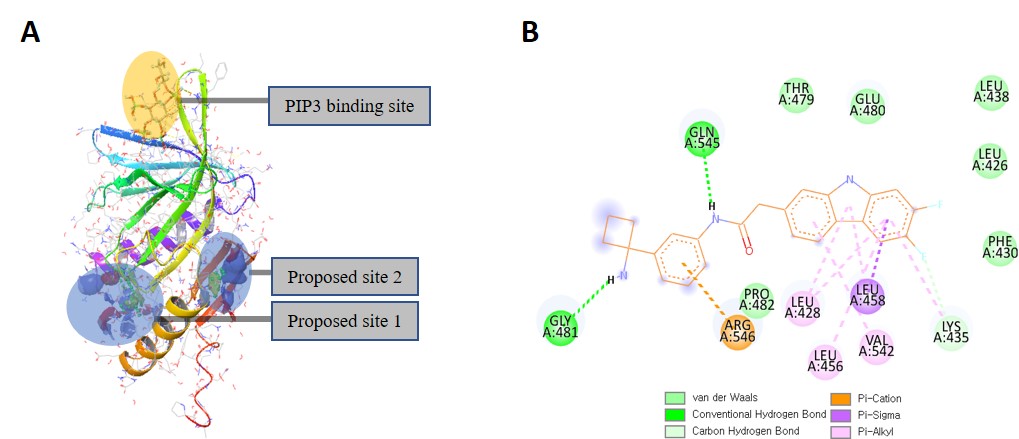


**Supplementary Figure 2.** **(A)** The binding pockets of the PH domain of PDK1 were identified using the Sitemap module in Maestro. The co-crystal ligand (PIP_3_) is shown in a stick model, and the protein structure is represented by the secondary structure. Sitemap proposed two potential binding sites, which are shown with blue (hydrogen bond donor), red (hydrogen bond acceptor), and yellow (hydrophobic) contours. The proposed site 1 had a larger volume than site 2; therefore, we used site 1 for the docking study of CU05-1189. **(B)** Two-dimensional diagram of the interaction between CU05-1189 and the PH domain of PDK1. Interacting residues are shown as spheres and interaction types are represented by different contours. The orange line indicates CU05-1189. (Figure prepared using Discovery Studio Visualizer)


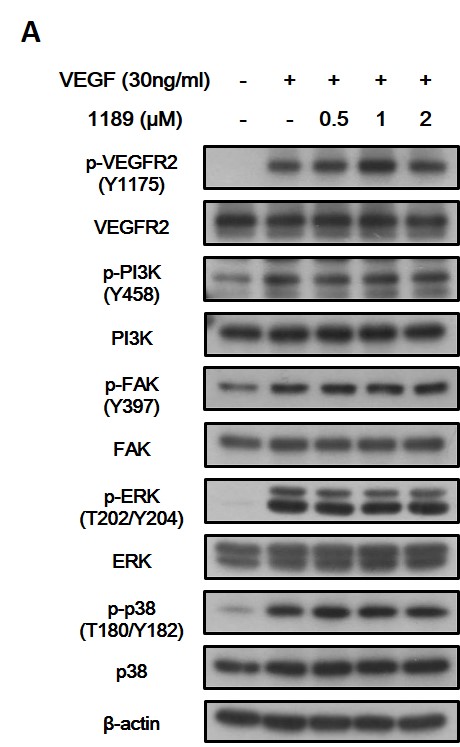


**Supplementary Figure 3.** **(A)** Western blot analysis of VEGF-stimulated VEGF receptor 2 signaling pathway activation after CU05-1189 treatment. VEGF receptor 2, PI3K, FAK, ERK, and p38 phosphorylation, as well as the overall levels of each protein, were examined after exposing HUVECs to CU05-1189 for 1 h, followed by 10 min of VEGF (30 ng/ml) stimulation.


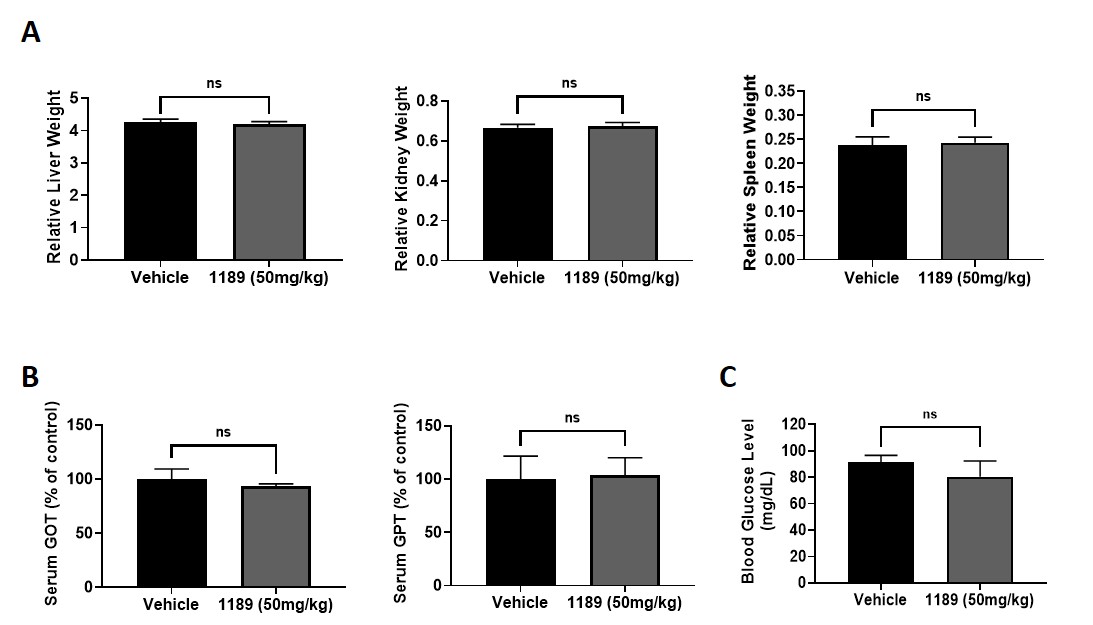


**Supplementary Figure 4. (A)** The relative organ weights for the liver, kidney and spleen in xenograft mice following oral administration of either the vehicle or CU05-1189 at a 50mg/kg dose. **(B)** Serum GOT and GPT levels. **(C)** Blood glucose level. Each value is presented as the mean±SEM, n=5-6 (ns = not significant).

## Supplementary Tables

**Supplementary Table 1. Pharmacokinetic Study of CU05-1189 in Sprague-Dawley Rats**

| Parameter (unit) | CU05-1189 | |
| --- | --- | --- |
|  | **i.v. (1 mg/kg)** | **p.o. (5 mg/kg)** |
| AUC_(0-_*_t_*_)_ (ng/ml·h) | 22422.2±633.6 | 76489.5±19565.2 |
| AUC_(0-∞)_ (ng/ml·h) | 51405.3±4822.2 | 97477.8±33416.6 |
| *C*_max_ (ng/ml) | 12115.9±1118.6 | 3428.7±1243.1 |
| *T*_max_ (h) |  | 6.0 |
| *T*_1/2_ (h) | 31.2±3.9 | 20.1±6.1 |
| *F* (%) |  | 68 |
